# Experimental Investigation of Thermal Conductivity of Selected 3D-Printed Materials

**DOI:** 10.3390/ma18173950

**Published:** 2025-08-22

**Authors:** Maria Tychanicz-Kwiecień, Sebastian Grosicki, Marek Markowicz

**Affiliations:** 1 Faculty of Mechanical Engineering and Aeronautics, Rzeszów University of Technology, al. Powstańców Warszawy 12, 35-959 Rzeszów, Poland; sebogr@prz.edu.pl; 2 Doctoral School of the Rzeszów University of Technology, Rzeszów University of Technology, al. Powstańców Warszawy 12, 35-959 Rzeszów, Poland; m.markowicz@prz.edu.pl

**Keywords:** thermal conductivity, 3D printing, PLA, ABS, PET-G, guarded heat flow method

## Abstract

This study presents the results of experimental studies on the thermal conductivity of specimens made from selected pure polymer filaments manufactured with the use of FFF 3D-printing technology. The tested samples were made of polylactic acid (PLA), polyethylene terephthalate glycol (PET-G), and acrylonitrile butadiene styrene (ABS). In particular, the effects of the infill patterns and infill density on the tested samples were examined in order to characterize the influence of these parameters on the materials’ effective thermal conductivity. Honeycomb and grid infill patterns of the tested samples with infill densities of 40%, 60%, 80%, and 100% were examined. The influence of temperature on thermal conductivity was studied as well. Thermal conductivity was measured using the guarded heat flow method, according to the ASTM E1530 standard within the defined temperature ranges of 20–60 °C for ABS and PET-G and 20–50 °C for PLA material. Samples of the tested materials were manufactured with the use of the Fused Filament Fabrication method (FFF), and filaments with a uniform black color were used. The obtained results were analyzed in terms of thermal conductivity variation after samples’ infill pattern and infill density modifications, which provides extended thermal property characterization of the polymeric filaments adopted for 3D printing.

## 1. Introduction

With technological development, 3D printing has brought new insights about reducing processing time and streamlining the design-to-production stage with confined material consumption in the long term, with regard to conventional manufacturing technologies. Among the many advantages of additive manufacturing processes, one of the greatest benefits is the ability to fabricate lightweight prototypes and components of an arbitrary shape with high precision from a previously generated CAD model. In comparison with traditional material processing techniques, such as material injection or extrusion, where components are characterized by complete material filling, 3D-printing technology allows for obtaining products with incomplete material filling, which results in significantly less material being used and reduced production costs. In the field of 3D-printing techniques, various methods are incorporated, including SLS (Selective Laser Sintering), SLA (Stereolithography), FFF (Fused Filament Fabrication), and many others [1,2,3]. For broadly defined engineering and industry applications, the FFF process is frequently selected due to its operation simplicity, general availability, as well as favorable economic and ecological considerations [1]. The principle of this method is based on the extrusion of a thin portion of melted filament layer by layer through a heated nozzle in order to obtain a complete part [4,5]. In the FFF process, various thermoplastic materials and polymers are used as filaments, including biodegradable components, such as PLA as well as ABS [6], PET-G [7,8], and many others. The application of rapid prototyping techniques is widespread and concerns the mechanical engineering, automotive industry, aeronautics, medicine, building [9], as well as the thermal energy industry. Current studies concerning 3D-printed products and prototypes are primarily centered on determining and improving the mechanical properties of the final components due to quite significant damage susceptibility in comparison to conventional manufacturing methods. However, apart from the assessment of material strength performance, comprehensive and exact characterization of 3D-printed components should also consider thermal properties. The applicability of 3D-printing technology, especially in the heat engineering industry, requires thorough thermal characterization of the materials used in order to ensure that certain thermal requirements are met [10,11]. Thermal performance evaluation is primarily based on the determination of the thermal conductivity parameter, which is a critical material property related to the ability to transfer heat via conduction in solid materials [12]. Other important thermal parameters are the specific heat and thermal diffusivity of the material [13]. The purpose of thermal conductivity investigations for materials used in 3D printing is to provide lacking supplementary data regarding the fundamental material properties of commonly applied filament materials. The literature shows that data regarding the thermal conductivity of 3D-printed materials and filaments are rather dissipated, constrained, or even unknown, as was noted by Bute et al. [1]. Polymer filaments used in 3D printing exhibit low thermal conductivity resulting from reasonably low atomic density, advanced internal crystal structure, and many other factors, as is remarked on in [12]. The low thermal conductivity of pure polymer filaments, and, thus, in the finished 3D-printed components, is a key drawback, which limits their broader range of applications, particularly in thermal energy and heat recovery systems. The literature shows that a primary research scope in the field of the thermal properties of materials employed in 3D printing includes modes of materials for thermal conductivity augmentation. One of the promising approaches for a thermal conductivity enhancement in the polymer filaments used in 3D printing is to use highly conductive materials as additives to modify the internal structure of the material, creating a polymer composite. In order to increase the thermal conductivity of polymeric filaments, the base material structure is reinforced with micro-powders of copper, aluminum, bronze, and other metal particles or carbon fibers [14,15]. Tsekmes et al. [16] reviewed the thermal conductivity of polymeric composites in terms of depicting methods of thermal conductivity enhancement by combining base polymer materials with highly conductive metal filler particles of various sizes. In the studied literature, it was noted that PLA is very frequently applied as a base filament material in 3D printing [4,10,17,18,19]. Laureto et al. [17] investigated the thermal properties of various metal–polymer composites as filaments in FFF 3D-printing technology. The effect of the presence of various metal particles on effective thermal conductivity was studied in detail. Also, the effects of material microstructure and printing parameters were analyzed in terms of thermal conductivity assessment. Sonsalla et al. [5] investigated the influence of 3D printer settings, including layer height, infill density, nozzle diameter and print speed, on the thermal conductivity of ABS material. Among other results, it was found that the increase in infill density yielded an increase in thermal conductivity. Ibrahim et al. [20] studied the effect of continuous carbon fiber addition as a reinforcement of 3D-printed polymer composites on thermal properties. Experimental and analytical characterization of the orientation and volume fraction of fibers on the effective thermal conductivity was carried out. In turn, in [18], an experimental and analytical investigation into the effective thermal conductivity of 3D-printed polymer composites reinforced with continuous metal wires was presented. The study aimed to provide insight into the impact of wire volume fraction, printing direction and matrix materials on composites’ thermal performance. Rodriguez et al. [10] presented experimental measurements of the thermal conductivity of seven distinct materials fabricated via the FFF method. The investigation involved pure PLA and ABS materials with 100% infill density as well as thermoplastic polyurethane (TPU), PEEK, ULTEM and materials reinforced with metal particles. Thermal conductivity was measured using the guarded heat flow method. The analysis highlighted the importance of determining the influence of the metal particle concentration and filler type on boosting the thermal performance of polymer composites adopted in 3D printing. Parmaksız et al. [21] experimentally studied the mechanical and thermal properties of seven filament material samples of variable thickness. In the range of materials studied, pure and reinforced PLA as well as ABS and TPU materials were selected. Thermal conductivity was measured with the use of a KD2 Pro thermal conductivity device. The variation in the thermal conductivity of materials with layer thickness was obtained. Tychanicz-Kwiecień et al. [4] presented a thermal performance investigation of PLA samples with a lattice infill pattern, creating closed air cells of distinct dimensions. Experimentally obtained effective thermal conductivity was verified via a numerical study of coupled heat transfer for certain sample geometric configurations. The analysis showed that for low-density samples, heat transfer is dominated by radiation along the surfaces. It was also found that effective thermal conductivity is not affected by the heat flow direction.

The explicit majority of the reported literature studies concerns the increase in the thermal conductivity of polymeric composites itself as a starting material for further material processing in 3D-printing technology. On the other hand, when considering final products manufactured using 3D printing, it was proved that thermal conductivity may be influenced by the shape and configuration of 3D-printed component filling, and, therefore, thermal conductivity can also be modified by altering 3D-printed components’ internal structure by decreasing sample infill density. Therefore, for thermal properties’ characterization, it is crucial to specify the temperature dependence of thermal conductivity while altering sample infill density. Once the thermal behavior of materials is known, the 3D-printing process can be scheduled in a way to minimize material consumption while maintaining the desired thermal characteristics of the final 3D-printed component within the intended operating temperature range. Nevertheless it is worth considering that the thermal properties of 3D-printed components made from polymer filaments may change due to anisotropic properties of the base material [22] and depend on material type, material porosity, presence of various additives, filament fiber orientation and heat flow direction relative to printing direction [10].

In the present work, we attempted to identify the impact and extent to which the modification of a sample’s internal structure can have on the effective thermal conductivity of 3D-printed components. Therefore, in this study, the results of experimental measurements of the thermal conductivity of PLA, PET-G and ABS polymer filaments fabricated via FFF 3D-printing technology are presented. The main objective of this study was to determine the influence of the sample infill density, infill pattern and temperature on the effective thermal conductivity of selected polymer materials. The scope of the presented investigation considered the variation in sample infill density, infill pattern and measurement temperature. All samples of the studied materials were fabricated in a wide range of infill densities of 40, 60, 80 and 100%. Also, all samples of each tested material for all infill densities were prepared in grid and honeycomb infill pattern variants. The thermal conductivity of each sample of tested material with certain infill pattern and infill density was measured within five distinct temperature ranges of 20, 30, 40, 50 and 60 °C For samples made of PLA, the maximum measurement temperature was reduced to 50 °C due to noticeable sample deformation at temperatures close to 60 °C. Thermal conductivity was measured using the guarded heat flow method, according to the ASTM E1530 standard, and for each established temperature, the thermal conductivity was determined three-fold. It was found that supreme thermal conductivity occurred for samples of 100% infill density for all investigated materials. Also, thermal conductivity varied slightly as a function of temperature, and the variation was similar for all investigated materials. The highest values of the thermal conductivity coefficient were recorded for a temperature of 50 °C. In terms of the infill pattern, it was observed that the thermal conductivity obtained for samples of the honeycomb infill pattern were considerably higher than the results obtained for the grid infill pattern.

## 2. 3D-Printing Technological Parameters and Sample Specification

**Figure 1:**
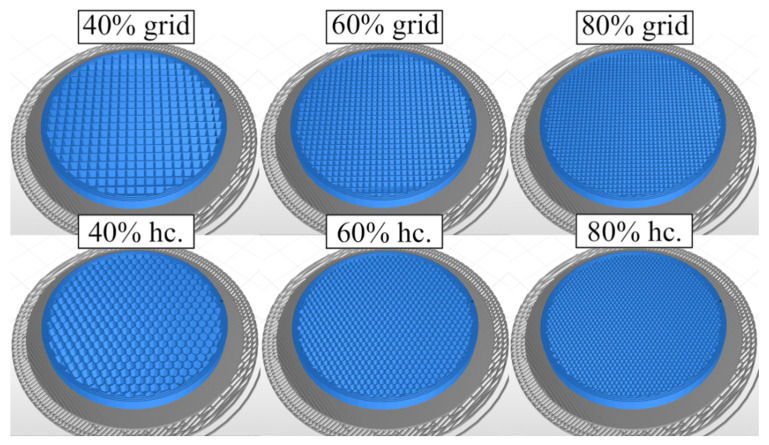
The model of cross-sections of investigated samples with grid and honeycomb infill patterns of variable infill densities.

**Figure 2:**
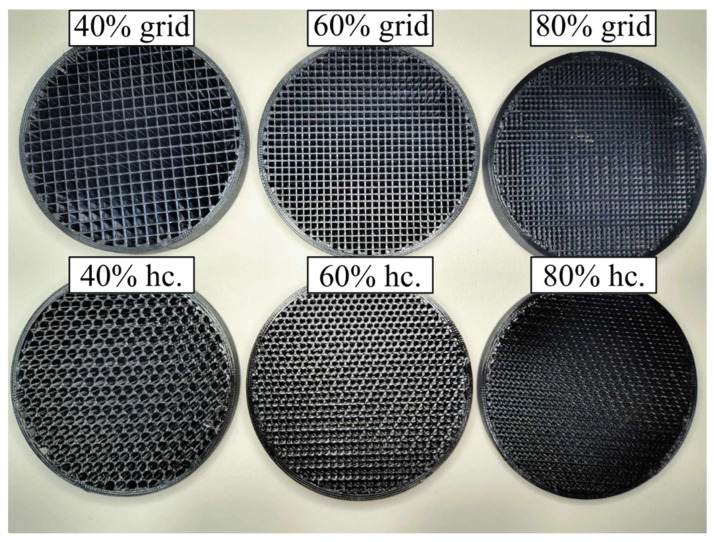
The real view of cross-section of investigated samples with grid and honeycomb infill patterns of variable infill densities.

In the present work, experimental investigations are carried out into the thermal conductivity coefficient of elements fabricated using 3D-printing technology. Disc-shaped test samples with a diameter of 50.8 mm and a height of 8 mm printed using FFF (Fused Filament Fabrication) technology were prepared. CAD models were developed using the Autodesk Inventor Professional software (Autodesk Inc, San Rafael, CA, USA). The ZORTRAX M300 Dual 3D printer (ZORTRAX, Olsztyn, Poland) and the manufacturer’s Z-Suite software (Slicer, ZORTRAX, Olsztyn, Poland) were used to make the research models. ZORTRAX is also the manufacturer of the materials used (Z-PETG, Z-PLA, Z-ABS). All models were made with the same nozzle diameter of 0.4 mm and a layer thickness of 0.15 mm. The default operating parameters dedicated to the material and printer model were used. For PLA, PET-G and ABS materials, the following parameters were applied, respectively: extrusion temperatures: 207 °C, 230 °C and 275 °C; platform temperatures: 30 °C, 40 °C and 80 °C; and printing speeds: 33 mm/s, 60 mm/s and 60 mm/s. The influence of measurement temperature, infill pattern and infill density was studied for three commonly used 3D-printing filaments. The parameters studied and test sample specification are presented in Table 1. The exemplary model and real view of the cross-sections of the investigated samples are presented in Figure 1 and Figure 2. Samples of all tested materials were manufactured from filaments of uniform black color, as presented illustratively in Figure 2.

## 3. Measurement Methodology

**Figure 3:**
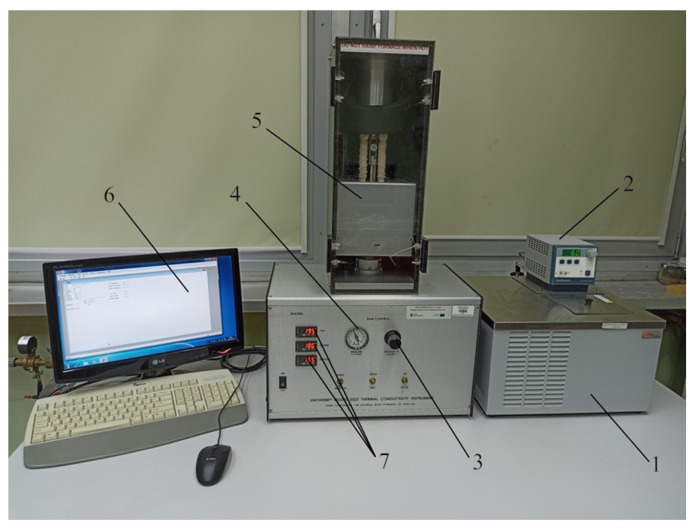
The view of the measuring station (Unitherm TM 2022, TA Instruments, New Castle, DE, USA): 1: ultrathermostat,.2: temperature regulator, 3: pressure regulator, 4: manometer, 5: guard heaters, 6: computer, 7: PID controller.

**Figure 4:**
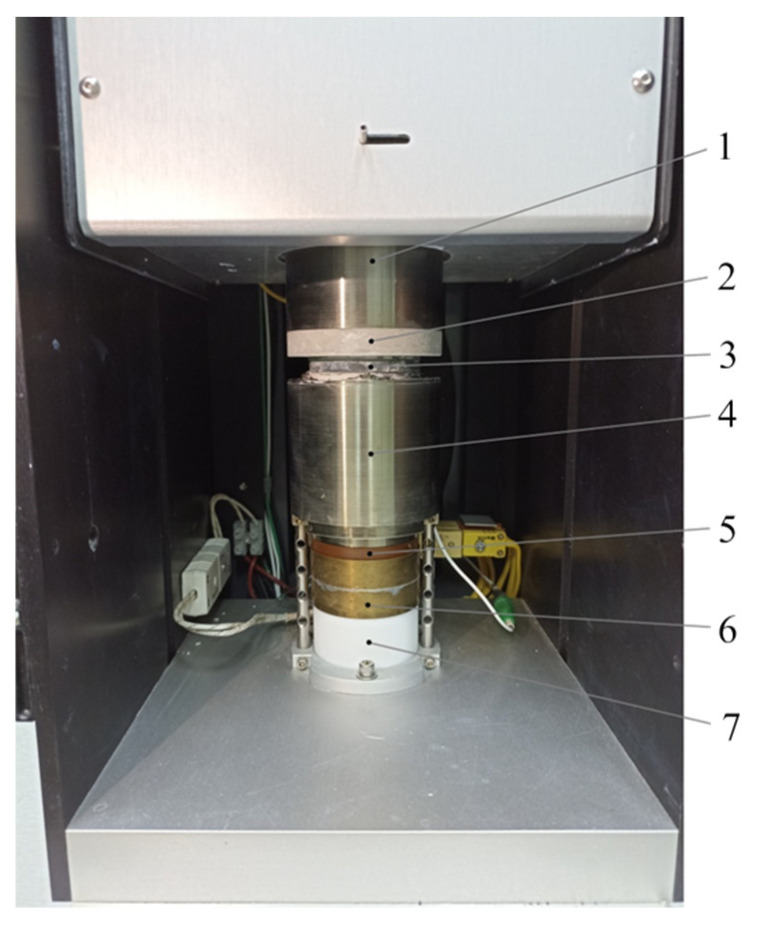
The view of the measuring section (Unitherm TM 2022, TA Instruments, New Castle, DE, USA): 1: upper radiator, 2: upper plate, 3: tested sample, 4: lower plate, 5: heat flux density meter, 6: bottom heater, 7: heat sink.

Thermal conductivity was measured according to the ASTM E1530 guarded heat flow method. Measurements were conducted according to [23], using the Unitherm ^TM^ 2022 device from Anter (currently TA Instruments, TA Instruments, New Castle, DE, USA). Figure 3 shows a general view of the test station, and Figure 4 shows the measurement section.

**Figure 5:**
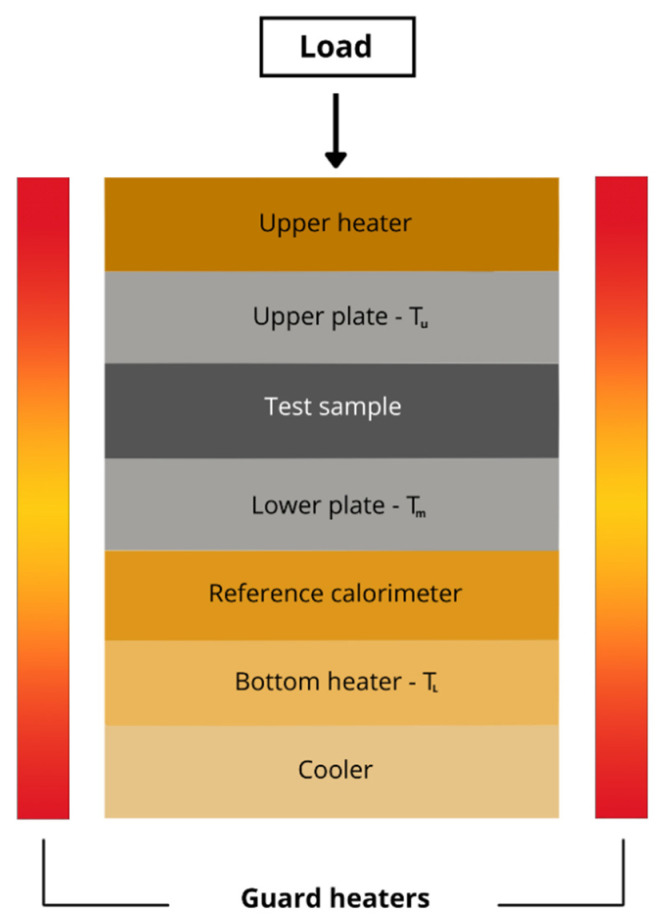
Test section scheme.

The test sample of the tested material is held under the reproducible compressive load between two polished surfaces (lower and upper plate), each controlled at a different temperature. The lower contact surface is a part of the calibrated heat flux sensor (see Figure 5). As heat flows from the upper surface through the sample to the lower surface, an axial temperature gradient is established in the stack. By measuring the temperature difference across the specimen with the output from the heat flux sensor, the thermal conductivity of the sample can be determined when the thickness is known. Guard heaters are placed outside the measuring section, for which the power is adjusted to limit heat losses from the side surface of the sample. A description of the experimental set-up as well as the thermal conductivity measurement specification are presented in detail in [4].

During the measurement, the thermal resistance of the sample R_s_ was measured, according to the equation:

(1) $$R_{s}=\frac{T_{u}-T_{m}}{Q}-R_{int}=\frac{T_{u}-T_{m}}{N\cdot \left(T_{m}-T_{L}\right)}-R_{int}=F\left(\frac{{∆T}_{s}}{{∆T}_{r}}\right)-R_{int}$$

where:

$R_{s}$: thermal resistance of the sample, m^2^ K/W;

$T_{u}$: temperature of the upper plate, K;

$T_{m}$: temperature of the lower plate, K;

$T_{L}$: temperature of the bottom heater, K;

Q: heat flux through the test sample, W/m^2^;

N: reference calorimeter heat transfer coefficient, m^2^/W;

$F\left(\frac{T_{u}-T_{m}}{T_{m}-T_{L}}\right)$: function determined during instrument calibration;

${∆T}_{s}=(T_{u}-T_{m})$;

${∆T}_{r}={(T}_{m}-T_{L})$;

$R_{int}$: total interface resistance between specimen and surface plates, m^2^ K/W.

The heat conduction coefficient was determined from the following relationship:

(2) $$R_{S}=\frac{d}{k},$$

where:

d: sample thickness, m;

k: thermal conductivity coefficient, W/mK.

All samples were covered on both sides with thermal paste before being placed in the measuring section. The coefficient of thermal conductivity was measured under steady-state conditions. After each measurement, the measuring stack was disassembled and the sample was removed. Then, the sample was again covered with the thermally conductive paste and placed in the measuring section.

The experimental investigation of thermal conductivity was scheduled in a way to conduct measurements three-fold for each sample of tested material of certain infill density and infill pattern. The temperature ranges, at which the thermal conductivity of the tested samples was planned to be determined, referred to the temperature set on the guard heaters. During experimental measurements, the temperature gradient established on the test stack was ∆T = 30 °C This indicates that the upper heater temperature was 15 °C higher and the cooler temperature was 15 °C lower than the defined temperature value. Therefore, the exact temperature values presented in this study corresponded to the average temperature measured for the test sample via a temperature sensor integrated into the measuring section. The laboratory room was air-conditioned, and the air temperature was maintained at a constant value of around 22 °C.

## 4. Results

Table 2, Table 3, Table 4 and Table 5 present the measured values of the thermal conductivity of the investigated samples for the materials studied. The experimental results presented below referred to the average thermal conductivity obtained from three experiments conducted for each average measured temperature. The measured values of thermal conductivity were provided with their total uncertainty, which was estimated according to the procedure presented in [24]. Table 2, Table 3 and Table 4 present the results obtained for PLA, ABS and PET-G materials of 40, 60 and 80% infill density with grid and honeycomb infill patterns, respectively. Table 5 contains a comparison of the measured thermal conductivity with measured temperatures for all investigated materials of 100% infill density.

The figures below present the experimental results of thermal conductivity measurements obtained for selected infill patterns and infill densities as a function of the average temperature measured for the test sample. In order to increase transparency of the results, both investigated infill patterns were presented separately for each material studied.

### 4.1. PLA Material

**Figure 6:**
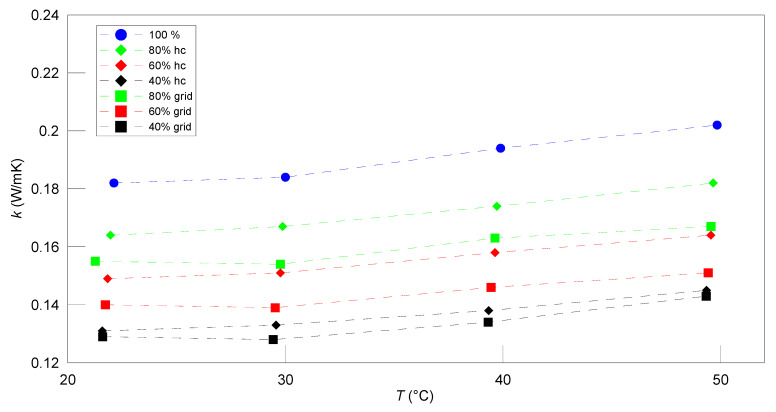
Experimental results of PLA thermal conductivity measurements as a function of temperature.

Figure 6 presents the result specification of thermal conductivity measurements as a function of temperature measured on the test samples made of PLA. The results were presented collectively for all selected infill patterns and infill densities. The border measurement temperature for PLA was reduced to 50 °C due to noticeable sample deformation at temperatures close to 60 °C. The distribution of thermal conductivity as a function of temperature exhibited slight and steady increases, observed for all investigated infill density and infill pattern variants. It was noticed that the thermal conductivity of the pure sample with 100% infill density was supreme among all investigated variants and increased almost linearly by 10% in the measured temperature range of T = 22.12−49.84 °C. In terms of sample infill pattern, it was found that the thermal conductivity obtained for samples with honeycomb infill pattern was higher within the entire range of measured temperatures in comparison to the results obtained for samples with the grid infill pattern. It was also observed that for both investigated infill patterns, the measured thermal conductivity decreased with an infill density decrease. For samples with a honeycomb infill pattern of 80% infill density, the relative difference in thermal conductivity with respect to the grid infill pattern was in the range of 5.5–9.5%; for samples of 60% infill density, thermal conductivity varied in the range of 6–7.5%; and for samples of 40% infill density, the relative difference between the thermal conductivity obtained for the honeycomb and grid infill patterns was the lowest and ranged between 1.5% and 4%.

In order to present the results more explicitly and profoundly, we decided to separate the results obtained for the honeycomb and grid infill patterns, and the analysis of thermal conductivity measurements was carried out as a function of infill density. The results are presented below.

**Figure 7:**
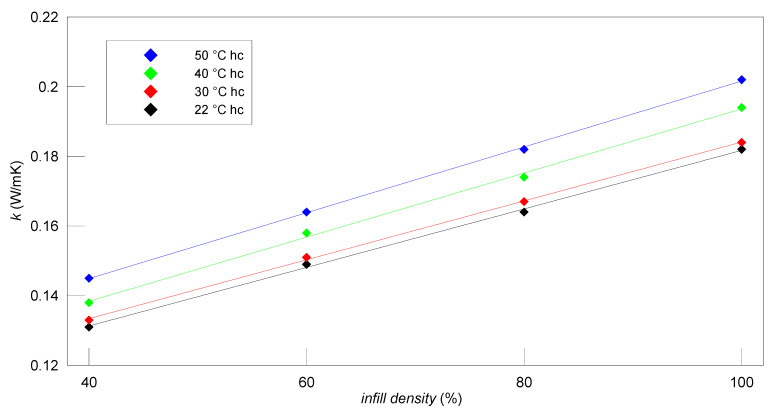
Experimental results of PLA thermal conductivity measurements as a function of infill density for honeycomb infill patterns.

Figure 7 presents the results of thermal conductivity measurements as a function of infill density for PLA samples with honeycomb infill patterns in the measured temperature ranges. It was found that the distribution of thermal conductivity for all measured temperature and infill density ranges can be successfully approximated using a linear function with the determination coefficient in a range of $R^{2}$ = 0.998−0.999 achieved for the whole investigated infill density variants. Therefore, the results were presented with a linear approximation function of the measured data parameters. The obtained results proved the previous observation that thermal conductivity within the entire uniform infill density ranges is the highest at the maximum measured temperature. The analysis of the relative decrease in thermal conductivity with decreasing infill density reflects that in reference to samples of 100% infill density, the thermal conductivity of samples with 80% infill density was decreased by 10%. According to samples with 60% infill density, thermal conductivity was decreased by around 18%, and for samples of 40% infill density, thermal conductivity was decreased by almost 30% in all measured temperature ranges.

**Figure 8:**
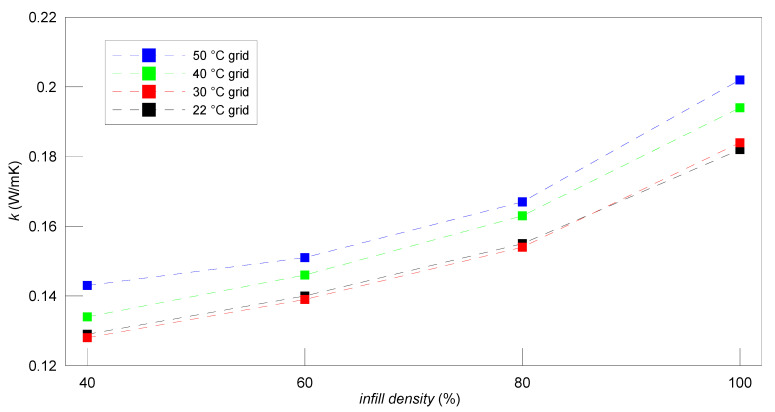
Experimental results of PLA thermal conductivity measurements as a function of infill density for grid infill patterns.

Figure 8 presents the results of thermal conductivity measurements as a function of infill density for PLA samples with grid infill patterns in the measured temperature ranges. It can be denoted that for samples with 40%, 60% and 80% infill densities, thermal conductivity measured at T = 22 °C was higher than that obtained at T = 30 °C, which is the opposite effect to that expected. For samples with 100% infill density, with increasing temperature, an increase in thermal conductivity is observed. Also, the increase in thermal conductivity with the infill density increase deviates to some extent from the linear one, particularly between infill densities of 80% and 100%.

### 4.2. PET-G Material

**Figure 9:**
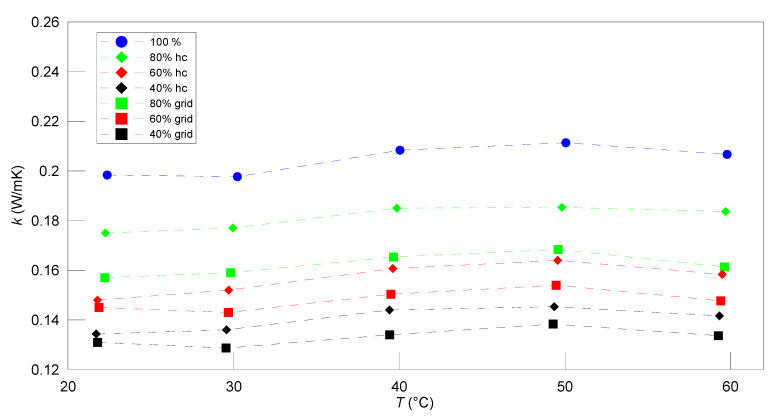
Experimental results of thermal conductivity measurements as a function of temperature for PET-G.

Figure 9 presents the consolidated results of PET-G thermal conductivity measurements as a function of temperature for both investigated infill patterns and infill densities. Due to the increased operating temperature range for PET-G and ABS, the maximum measurement temperature was elevated to T = 60 °C.

As predicted, the maximum measured thermal conductivity within the entire average measured temperature ranges was reached for the pure sample with 100% infill density. It is worth emphasizing that in certain temperature ranges, the measured thermal conductivity either remained the same or even decreased. For the sample with 100% infill density, the thermal conductivity at T = 22.36 °C and T = 30.22 °C was equal to k = 0.198 W/mK (stdv = 0.002 and stdv = 0.001, respectively), and within the temperature range of T = 50.05 °C–59.78 °C, the thermal conductivity decreased from k = 0.211 W/mK to k = 0.207 W/mK. A drop in the thermal conductivity at a temperature close to 60 °C was observed for all investigated infill patterns and infill densities. A slight increase in thermal conductivity was noticed only within the measured temperature range of T = 30.22 °C–50.05 °C, reaching its maximum at the approximate measured temperature  T = 50 °C.

As far as the infill pattern is concerned, the thermal conductivity obtained for samples of honeycomb infill patterns was higher in comparison to samples with grid infill patterns, which was also observed for PLA material. For samples with 80% infill density, the thermal conductivity obtained for honeycomb infill patterns was about 10% higher than that obtained for grid infill patterns within the average measured temperature ranges. When the sample infill density decreased to 60% and 40%, the relative difference in the thermal conductivity for honeycomb and grid infill patterns was gradually reduced to approximately 7% and 5% within the average measured temperature ranges, respectively.

**Figure 10:**
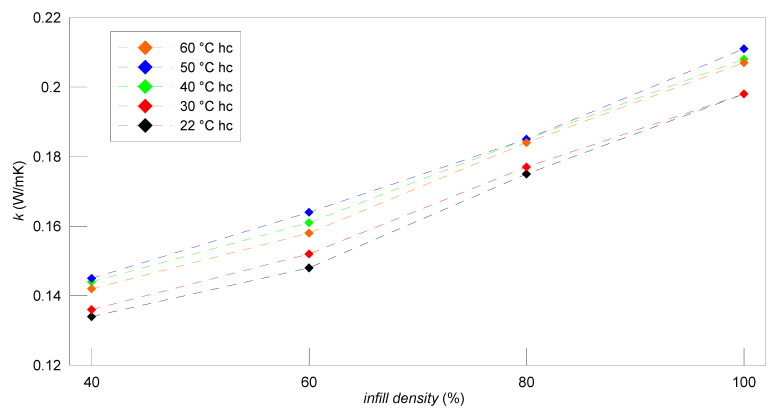
Experimental results of PET-G thermal conductivity measurements as a function of infill density for honeycomb infill patterns.

The results of thermal conductivity measurements as a function of infill density for PET-G samples with honeycomb infill patterns in the average measured temperature ranges are presented in Figure 10. The steady increase in thermal conductivity with the infill density increase is evident. However, in contrast to the results obtained for PLA samples, it was found that the thermal conductivity of PET-G samples is not an explicitly linear function of the infill density within the entire established temperature ranges. For approximate measured temperature T = 50 °C, the thermal conductivity distribution is the most similar to the linear one. However, as the measurement temperature decreases, the thermal conductivity distribution progressively diverges from the linear function. Moreover, for samples with 80% infill density within the measured temperatures T = 40 °C–60 °C, the thermal conductivity nearly coincided. The thermal conductivity imposition is also observed for samples with 100% infill density at temperatures of T=22 °C T = 40 °C and T = 60 °C as well as for samples with 40% infill density at temperatures of T = 40 °C–50 °C.

**Figure 11:**
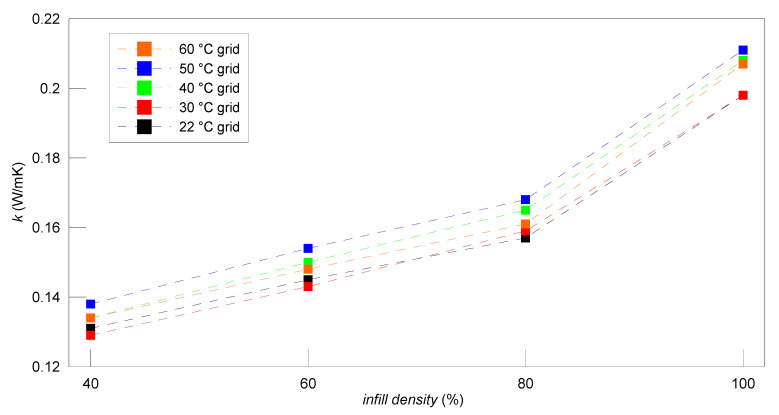
Experimental results of PET-G hermal conductivity measurements as a function of infill density for grid infill patterns.

Figure 11 presents the experimental results of PET-G thermal conductivity measurements as a function of infill density for samples with grid infill patterns in the average measured temperature ranges. The rapid increase in thermal conductivity for samples of infill density in ranges of 80% and 100% for all measured temperatures is clearly noticeable. The relative difference between the thermal conductivity obtained for samples with 100% and 80% infill density within the entire measured temperature ranges reached over 20%. Moreover, for samples with 40% infill density at temperatures T = 40 °C and T = 60 °C, the thermal conductivity overlapped, which was also noticed for samples with 100% infill density at temperatures of T = 22 °C and T = 30 °C in addition, for samples with 40% and 60% infill density, the thermal conductivity measured at T = 22 °C was higher than that obtained at T = 30 °C.

### 4.3. ABS Material

**Figure 12:**
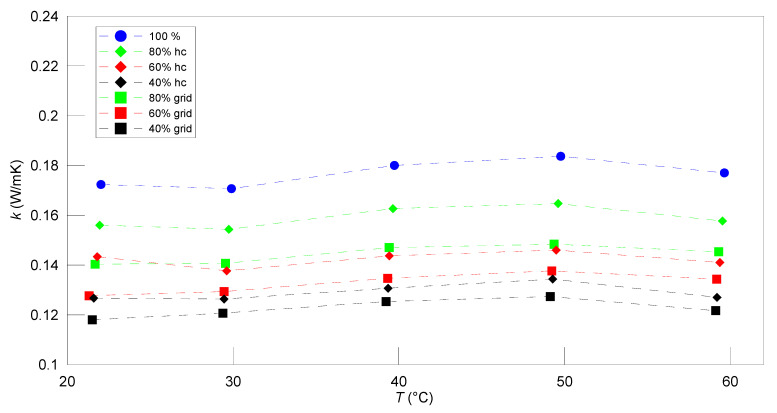
Experimental results of thermal conductivity measurements as a function of temperature for ABS.

The experimental results of ABS thermal conductivity measurements as a function of temperature for both investigated infill patterns and infill densities are presented in Figure 12. It follows that, likewise, for PLA and PET-G materials, supreme thermal conductivity values were achieved for samples of 100% infill density for the entire established temperature ranges. For both investigated infill patterns and infill densities, the measured thermal conductivity slightly increased as a function of temperature; however, at temperatures around 60 °C, the thermal conductivity decreased by approximately 5% at 50 °C for all infill densities and infill patterns. A similar effect was also observed for PET-G material. In a temperature range between T = 20 °C and 30 °C, the thermal conductivity remained nearly constant, and for samples of the honeycomb infill pattern with 60% infill density, the thermal conductivity decreased apparently at the exact measured temperatures T = 21.81 °C, and T = 29.62 °C. For all investigated infill density and infill pattern variants, the thermal conductivity was extreme at temperatures close to T = 50 °C. In order to assign the influence of the infill pattern on thermal conductivity, it was found that samples with the honeycomb infill pattern exhibited higher thermal conductivity than samples with the grid infill pattern throughout the entire measured temperature ranges.

**Figure 13:**
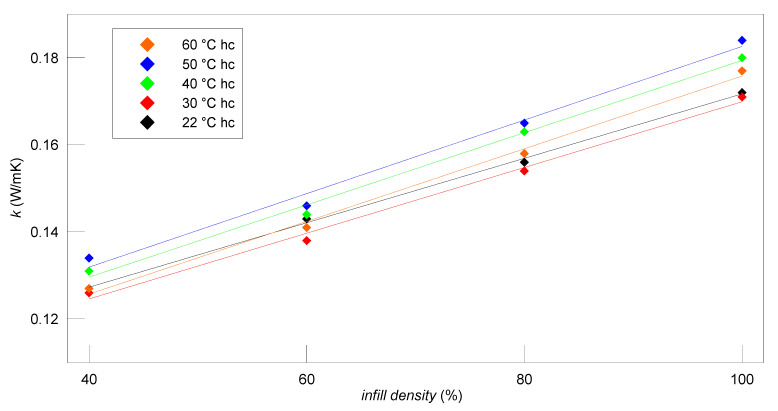
Experimental results of ABS thermal conductivity measurements as a function of infill density for honeycomb infill patterns.

Figure 13 presents the experimental results of ABS thermal conductivity measurements as a function of the infill density for honeycomb infill patterns in the average measured temperature ranges. From the presented results, it can be noticed that, likewise, for PLA material, for all measured temperatures, the thermal conductivity as a function of infill density can be approximated by a linear function with the determination coefficient ranging between $R^{2}$ = 0.989 and 0.998. However, the thermal conductivity variation and infill density variation are not completely obvious. It can be seen that for the sample of 60% infill density, the approximation function proceeded at a temperature of T = 22 °C with the intersecting line corresponding to a temperature of T = 60 °C. This indicates that the thermal conductivity for a given infill density does not increase evenly with increasing temperature.

**Figure 14:**
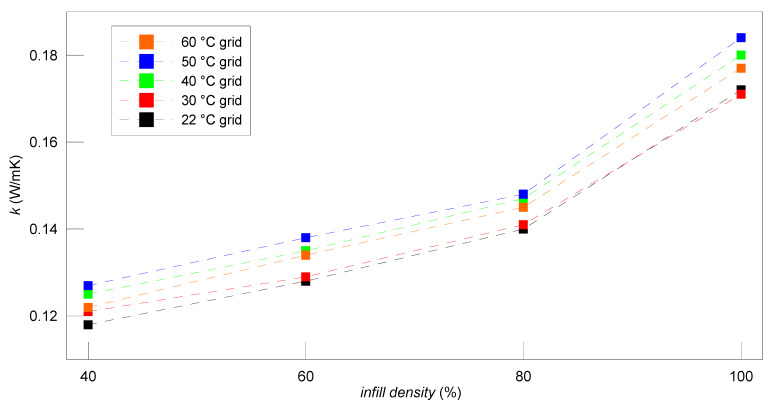
Experimental results of ABS thermal conductivity measurements as a function of infill density for grid infill patterns.

Figure 14 presents the experimental results of ABS thermal conductivity measurements as a function of the infill density for grid infill patterns for the average measured temperature ranges. Likewise, for PET-G and PLA materials, the thermal conductivity increased almost linearly for samples with 40%, 60% and 80% infill density, and a considerable increase in thermal conductivity can be observed for samples of infill density in ranges between 80% and 100% for all measured temperatures. The relative difference between the thermal conductivity obtained for samples with 100% and 80% infill density within the entire measured temperature ranges was in the range of 17.5–19.5%. For 60%, 80% and 100% infill densities, the thermal conductivity measured at T = 20 °C and T = 30 °C was approximately equal.

## 5. Conclusions

Experimental studies of the thermal conductivity coefficients of samples produced using 3D-printing technology were conducted. Three-dimensional-printing technology, particularly the FFF method, is a dynamically developing manufacturing method, gaining popularity due to the possibility of economically producing spare parts or device components, even under domestic conditions. However, according to the literature [8], even in printed elements with 100% infill density, as a result of the printing process, air spaces are created and enclosed in the sample volume, causing a decrease in the thermal conductivity of the material relative to the base material. In addition, the desire to speed up the printing process, reduce the weight of printed elements or reduce production costs means that 3D printer users often decide to lower the infill density below 100%, which additionally increases the spaces with air enclosed in the sample. With a decrease in the infill density below 100%, there is an additional need to choose the infill pattern. The aim of this work was to investigate the effect of the infill density and the infill pattern on the thermal conductivity coefficient. Moreover, due to the dependence of thermal conductivity on temperature, measurements were conducted for various temperature ranges. The test samples were made of three commonly used materials in terms of 3D printing using the FFF method (PLA, PET-G, ABS). Filaments of the same color were selected to eliminate the influence of dyes. When considering the reduced temperature range for PLA sample measurements, the general shape of the course of changes in the thermal conductivity coefficient with temperature is similar regardless of the material used. However, changes in the thermal conductivity coefficient as a function of the infill density for a given temperature are not as obvious. Regardless of the material and the infill pattern, the highest values of the thermal conductivity coefficient were recorded for a temperature of 50 °C, followed by 40 °C. In other cases, the selection of a given temperature to maximize the thermal conductivity coefficient for the entire range of infill densities is not clear. The least predictable in this respect was the PET-G material.

**Figure 15:**
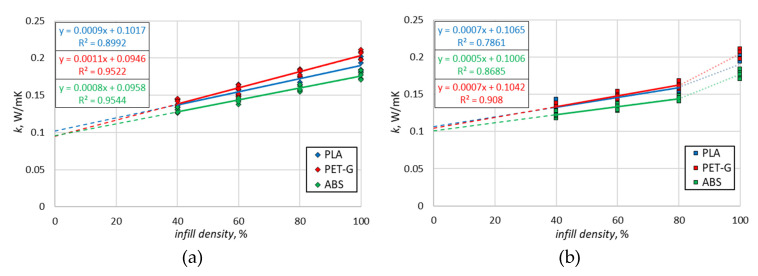
Experimental results of all samples tested as a function of infill density for average measured temperature ranges for: (**a**) honeycomb infill patterns, (**b**) grid infill pattern.

With decreasing infill density, the thermal conductivity coefficient also decreased, as expected. This is caused by the increase in the volume fraction of air enclosed in the sample volume (air has a lower thermal conductivity coefficient than the tested materials), which was also noticed in [17]. Figure 15 shows an approximation of the results with a linear function, depending on the infill pattern. The data included measurements taken over the entire established temperature range, which resulted in reduced values of the determination coefficient. Therefore, this graph can be treated as illustrative material. It was intended to provide a general comparison of the tested materials in the context of changes in the thermal conductivity coefficient as a function of infill density. Furthermore, the approximations were extended by a range of 0–40% infill density (dashed line). This extension was used only for visual purposes in order to better illustrate the trend, although the value of the 0% fill density has no physical sense. In the case of the grid infill pattern, the approximation was based on results corresponding to 40–80% of the infill density, and the approximation itself is characterized by a lower determination coefficient. An interesting observation is the different rate of decrease in the thermal conductivity coefficient with the infill density, depending on the material. Moreover, within a given material, this rate depends on the infill pattern. For both infill patterns, the smallest percentage decrease is characterized by samples made of ABS, while the largest percentage decrease is characterized by samples made of PET-G.

As far as the infill pattern for all temperatures and materials is concerned (Figure 15), the selection of the grid infill pattern was characterized by a significant decrease in the value of the thermal conductivity coefficient in relation to the corresponding honeycomb infill pattern (even by approximately 12% for PET-G at 60 °C). One unexpected effect was the sudden decrease in the thermal conductivity coefficient value for all samples between 100% infill density and 80% for the grid infill pattern (even by approximately 22% for the PET-G material), while for the honeycomb infill pattern, the changes in the entire range of infill density were approximately linear. The results for the 40–80% infill density for the grid infill pattern can also be approximated with a relatively high accuracy using a linear function. Based on these results, it can be concluded that the mere choice of the grid infill pattern causes a significant decrease in the value of the thermal conductivity coefficient.

Moreover, a surprising observation regarding the decrease in thermal conductivity obtained at T = 60 °C with respect to T = 50 °C for ABS and PET-G was exposed. The analysis provided the anticipation that the measurement temperature was near the glass transition temperature of the materials. In [13], the ABS glass temperature was denoted as 105 °C. According to [25], the PET-G glass transition temperature was identified as 80 °C. On the other hand, the PLA glass transition temperature determined in [26] was 65 °C. The temperature set on the upper heater was 15 °C higher than the temperature measured on the test sample; therefore, for border temperature measurements, the set temperature was around 75 °C. According to [10], the maximum secure operating temperature indicated for ABS material was limited to 81 °C and, for PLA, to 55 °C. It is also worth mentioning that due to the significant diversity of polymer material types, the glass transition temperature limit is not explicitly determined but is typically contained in certain temperature ranges. The thermal parameters, particularly the glass transition temperature, provided by manufacturers for filaments used in 3D printing often denote the critical temperature value. Further explicit analysis of the thermal behavior of ABS and PET-G at elevated temperatures requires the investigation and consideration of the materials’ mechanical properties as well.

The secondary scope of this investigation was to provide in-depth thermal properties’ characterization, with particular emphasis on the thermal conductivity of filaments used in 3D printing in a wide range of measured temperatures. As remarked upon in [13], due to the nature of the additive manufacturing process, where successive heating and cooling processes occur, both the mechanical and thermal properties of final 3D-printed components strictly depend on the heating and cooling temperatures and conditions. Therefore, in order to initially predict the thermal behavior of 3D-printed elements, thermal conductivity studies are of particular importance. The literature concerning thermal conductivity investigations of materials used in 3D printing is extensive; however, direct comparisons of the thermal conductivity of materials and samples made via 3D-printing technology are rather confined due to discrepancies in the scope of the research conducted and the selected types of materials tested.

Due to the rapidly developing and increasingly popular additive technology of 3D printing, further research is necessary on printed elements. Although the basic mechanical properties are provided by manufacturers on filament catalogue cards and researchers are also eager to supplement the list of these parameters with their publications, thermal parameters of filaments, and even more so of printed elements, are very rare. The research presented in this study was of a general nature, focusing on the main parameters of 3D-printed elements (material, infill density, infill pattern). The parameters of the 3D-printing process itself, for example, the printing speed and the printing temperature, were assumed as the default recommended by the manufacturer; however, further work is recommended in this field. In the near future, the authors plan to investigate other filament colors, as well as to adopt other 3D-printing materials and parameters, in order to assess their influence on thermal performance characterization.

## Figures and Tables

**Table 1:** Test sample specification.

Material	Temperature Set on Guard Heaters [°C]	Infill Pattern	Infill Density [%]
PLA PET-G * ABS *	20 30 40 50 60 *	Honeycomb (hc.)	40
			60
			80
		Grid	40
			60
			80
		---	100

* refers to the materials with maximum established temperature of 60 °C.

**Table 2:** Average values of the thermal conductivity coefficient for average measured temperatures for PLA.

PLA						
	40% grid		60% grid		80% grid	
	T ℃	k W/mK	T ℃	k W/mK	T ℃	k W/mK
1.	**21.60** **±** 0.25	**0.129** **±** 0.004	**21.73** **±** 0.27	**0.140** **±** 0.006	**21.27** **±** 0.66	**0.155** **±** 0.005
2.	**29.44** **±** 0.26	**0.128** **±** 0.004	**29,53** **±** 0.26	**0.139** **±** 0.005	**29.77** **±** 0.25	**0.154** **±** 0.005
3.	**39.32** **±** 0.25	**0134** **±** 0.005	**39.45** **±** 0.26	**0.146** **±** 0.005	**39.63** **±** 0.25	**0.163** **±** 0.005
4.	**49.34** **±** 0.26	**0.143** **±** 0.004	**49.43** **±** 0.25	**0.151** **±** 0.005	**49.56** **±** 0.26	**0.167** **±** 0.005
	**40% hc.**		**60% hc.**		**80% hc.**	
	T ℃	k W/mK	T ℃	k W/mK	T ℃	k W/mK
1.	**21.59** **±** 0.26	**0.131** **±** 0.005	**21.83** **±** 0.25	**0.149** **±** 0.005	**21.96** **±** 0.25	**0.164** **±** 0.006
2.	**29.57** **±** 0.25	**0.133** **±** 0.004	**29.77** **±** 0.25	**0.151** **±** 0.005	**29.87** **±** 0.25	**0.167** **±** 0.005
3.	**39.34** **±** 0.25	**0.138** **±** 0.004	**39.63** **±** 0.25	**0.158** **±** 0.005	**39.72** **±** 0.25	**0.174** **±** 0.005
4.	**49.35** **±** 0.25	**0.145** **±** 0.005	**49.55** **±** 0.25	**0.164** **±** 0.005	**49.66** **±** 0.25	**0.182** **±** 0.005

**Table 3:** Average values of the thermal conductivity coefficient for average measured temperatures for PET-G.

PET G						
	40% grid		60% grid		80% grid	
	T ℃	k W/mK	T ℃	k W/mK	T ℃	k W/mK
1.	**21.79** **±** 0.28	**0.131** **±** 0.004	**21.87** **±** 0.25	**0.145** **±** 0.005	**22.22** **±** 0.27	**0.157** **±** 0.005
2.	**29.54** **±** 0.26	**0.129** **±** 0.004	**29.68** **±** 0.25	**0.143** **±** 0.005	**29.82** **±** 0.25	**0.159** **±** 0.005
3.	**39.41** **±** 0.25	**0.134** **±** 0.004	**39.49** **±** 0.26	**0.150** **±** 0.005	**39.64** **±** 0.25	**0.165** **±** 0.005
4.	**49.28** **±** 0.25	**0.138** **±** 0.004	**49.47** **±** 0.25	**0.154** **±** 0.005	**49.59** **±** 0.25	**0.168** **±** 0.005
5.	**59.25** **±** 0.25	**0.134** **±** 0.004	**59.40** **±** 0.25	**0.148** **±** 0.004	**59.63** **±** 0.26	**0.161** **±** 0.005
	**40% hc.**		**60% hc.**		**80% hc.**	
	T ℃	k W/mK	T ℃	k W/mK	T ℃	k W/mK
1.	**21.70** **±** 0.27	**0.134** **±** 0.004	**21.77** **±** 0.25	**0.148** **±** 0.005	**22.25** **±** 0.25	**0.175 ±** 0.005
2.	**29.56** **±** 0.25	**0.136** **±** 0.004	**29.70** **±** 0.25	**0.152** **±** 0.005	**29.95** **±** 0.25	**0.177 ±** 0.005
3.	**39.40** **±** 0.25	**0.144** **±** 0.004	**39.61** **±** 0.25	**0.161** **±** 0.005	**39.85** **±** 0.25	**0.185 ±** 0.006
4.	**49.35** **±** 0.25	**0.145** **±** 0.005	**49.56** **±** 0.25	**0.164** **±** 0.005	**49.82** **±** 0.25	**0.185 ±** 0.006
5.	**59.32** **±** 0.25	**0.142** **±** 0.004	**59.49** **±** 0.25	**0.158** **±** 0.005	**59.70** **±** 0.25	**0.184 ±** 0.006

**Table 4:** Average values of the thermal conductivity coefficient for average measured temperatures for ABS.

ABS						
	40% grid		60% grid		80% grid	
	T ℃	k W/mK	T ℃	k W/mK	T ℃	k W/mK
1.	**21.51** **±** 0.27	**0.118** **±** 0.004	**21.32** **±** 0.28	**0.128** **±** 0.004	**21.69** **±** 0.25	**0.140** **±** 0.004
2.	**29.38** **±** 0.25	**0.121** **±** 0.004	**29.45** **±** 0.25	**0.129** **±** 0.004	**29.55** **±** 0.25	**0.141** **±** 0.004
3.	**39.22** **±** 0.25	**0.125** **±** 0.004	**39.32** **±** 0,25	**0.135** **±** 0.004	**39.41** **±** 0.25	**0.147** **±** 0.005
4.	**49.13** **±** 0.25	**0.127** **±** 0.004	**49.22** **±** 0.25	**0.138** **±** 0.004	**49.36** **±** 0.25	**0.148** **±** 0.005
5.	**59.10** **±** 0.25	**0.122** **±** 0.004	**59.17** **±** 0.25	**0.134** **±** 0.004	**59.29** **±** 0.25	**0.145** **±** 0.004
	**40% hc.**		**60% hc.**		**80% hc.**	
	T ℃	k W/mK	T ℃	k W/mK	T ℃	k W/mK
1.	**21.60** **±** 0.25	**0.127** **±** 0.004	**21.81** **±** 0.25	**0.143** **±** 0.005	**21.96** **±** 0.26	**0.156** **±** 0.006
2.	**29.45** **±** 0.25	**0.126** **±** 0.004	**29.62** **±** 0.25	**0.138** **±** 0.004	**29.75** **±** 0.25	**0.154** **±** 0.005
3.	**39.35** **±** 0.25	**0.131** **±** 0.004	**39.43** **±** 0.25	**0.144** **±** 0.004	**39.64** **±** 0.25	**0.163** **±** 0.005
4.	**49.28** **±** 0.25	**0.134** **±** 0.004	**49.49** **±** 0.26	**0.146** **±** 0.005	**49.60** **±** 0.25	**0.165** **±** 0.005
5.	**59.19** **±** 0.25	**0.127** **±** 0.004	**59.36** **±** 0.25	**0.141** **±** 0.004	**59.52** **±** 0.26	**0.158** **±** 0.005

**Table 5:** A comparison of measured thermal conductivity with measured temperatures for all investigated materials of 100% infill densities.

	PLA 100%		PET G 100%		ABS 100%	
	T ℃	k W/mK	T ℃	k W/mK	T ℃	k W/mK
1.	**22.12** **±** 0.25	**0.182** **±** 0.006	**22.36** **±** 0.25	**0.198** **±** 0.006	**22.03** **±** 0.26	**0.172** **±** 0.006
2.	**30.00** **±** 0.25	**0.184** **±** 0.006	**30.22** **±** 0.25	**0.198** **±** 0.006	**29.90** **±** 0.25	**0.171** **±** 0.005
3.	**39.89** **±** 0.25	**0.194** **±** 0.006	**40.04** **±** 0.25	**0.208** **±** 0.006	**39.73** **±** 0.25	**0.180** **±** 0.005
4.	**49.84** **±** 0.26	**0.202** **±** 0.006	**50.05** **±** 0.25	**0.211** **±** 0.006	**49.76** **±** 0.26	**0.184** **±** 0.006
5.	**---**	**---**	**59.78** **±** 0.31	**0.207** **±** 0.006	**59.63** **±** 0.26	**0.177** **±** 0.005

## Data Availability

The original contributions presented in this study are included in the article Material. Further inquiries can be directed to the corresponding authors.
